# Dermal γδ T Cells Do Not Freely Re-Circulate Out of Skin and Produce IL-17 to Promote Neutrophil Infiltration during Primary Contact Hypersensitivity

**DOI:** 10.1371/journal.pone.0169397

**Published:** 2017-01-12

**Authors:** Xiaodong Jiang, Chang Ook Park, Jenna Geddes Sweeney, Min Jae Yoo, Olivier Gaide, Thomas Seth Kupper

**Affiliations:** Harvard Skin Disease Research Center, Department of Dermatology, Brigham and Women’s Hospital, Harvard Medical School, Boston, Massachusetts, United States of America; CCAC, UNITED STATES

## Abstract

The role of mouse dermal γδ T cells in inflammatory skin disorders and host defense has been studied extensively. It is known that dendritic epidermal T cells (DETC) have a monomorphic γδ T cell receptor (TCR) and reside in murine epidermis from birth. We asked if dermal γδ cells freely re-circulated out of skin, or behaved more like dermal resident memory T cells (T_RM_) in mice. We found that, unlike epidermal γδ T cells (DETC), dermal γδ cells are not homogeneous with regard to TCR, express the tissue resident T cell markers CD69 and CD103, bear skin homing receptors, and produce IL-17 and IL-22. We created GFP^+^: GFP^−^ parabiotic mice and found that dermal γδ T cells re-circulate very slowly—more rapidly than authentic αβ TCR T_RM_, but more slowly than the recently described dermal αβ TCR T migratory memory cells (T_MM_). Mice lacking the TCR δ gene (δ^-/-^) had a significant reduction of 2,4-dinitrofluorobenzene (DNFB)-induced contact hypersensitivity (CHS). We created mice deficient in dermal γδ T cells but not DETC, and these mice also showed a markedly reduced CHS response after DNFB challenge. The infiltration of effector T cells during CHS was not reduced in dermal γδ T cell-deficient mice; however, infiltration of Gr-1^+^CD11b^+^ neutrophils, as well as ear swelling, was reduced significantly. We next depleted Gr-1^+^ neutrophils in vivo, and demonstrated that neutrophils are required for ear swelling, the accepted metric for a CHS response. Depletion of IL-17-producing dermal Vγ4^+^ cells and neutralization of IL-17 in vivo, respectively, also led to a significantly reduced CHS response and diminished neutrophil infiltration. Our findings here suggest that dermal γδ T cells have an intermediate phenotype of T cell residence, and play an important role in primary CHS through producing IL-17 to promote neutrophil infiltration.

## Introduction

γδ T cells represent a small fraction (1–5%) of the overall T cell population but are abundant in barrier tissues like skin [1]. Dendritic Epidermal T cells (DETC), expressing a distinctive invariant Vγ5/Vδ1 TCR, were thought to be the only γδ T cell population in murine skin and have been studied for decades for their function in wound repair, tumor surveillance and inflammation [2]. More recently, a distinct population of γδ T cells was identified in murine dermis. These dermal γδ T cells have polymorphic TCR repertoires distinct from DETC, and upon activation produce abundant IL-17 [3, 4]. It has been suggested that dermal γδ T cells represent an important source of IL-17 in murine skin and may initiate the pathogenesis of murine models of psoriasoform dermatitis [5–9].

Allergic contact dermatitis (ACD) is a common skin disease affecting 15–20% of the general population in the world [10]. The best accepted animal model of ACD is mouse contact hypersensitivity (CHS), which is a delayed-type immune response following skin contact with certain reactive chemicals called haptens. These chemicals, such as 2,4-dinitrofluorobenzene (DNFB), oxazolone, fluorescein isothianate (FITC) and trinitrochlorobenzene (TNCB), have a low molecular weight (<500 daltons), are highly reactive with proteins, and form hapten-carrier complexes to elicit adaptive immune responses. Importantly, this acute CHS assay measures a primary recall, and not a true memory immune response, as the sensitization and challenge are separated by only five days (not nearly enough time for authentic adaptive immune memory to develop). Our more recent studies have characterized a new model of murine CHS, which like human ACD, is mediated in large part by αβ TCR T_RM_ [11]. This type of CHS response can be triggered more than 100 days after sensitization [11]. However, whether γδ T cells also participate in the murine model of acute CHS is the subject of some debate. For example, using γδ T cell-deficient (TCR δ^-/-^) mice, one group did not observe any significant change of ear swelling after challenge [12], while another group found a markedly increased primary CHS response [13]. Recently, yet another group reported a strong reduction in ear swelling in TCR δ^-/-^ mice [14], the role of DETC in primary CHS was also evaluated by adoptive transfer of these cells to mice with spontaneous dermatitis, and by using mice with functionally defective DETC. Both studies showed that DETC appear to suppress the CHS response through an unknown mechanism [15].

The role of IL-17 in human allergic contact dermatitis was suggested by a study showing that approximately half of nickel-specific CD4^+^ T cell clones isolated from nickel-allergic patients produce IL-17 [16]. Direct evidence for the role of IL-17 in the murine acute CHS response was shown by using IL-17^-/-^ mice, in which a markedly suppressed ear swelling response was observed [17]. CD4^+^ Th17 cells have been regarded as one main source of IL-17 in CHS, although some subpopulations of CD8^+^ T cells have been implicated as well [18]. In recent years, γδ T cells have been identified as a major source of IL-17 in murine peripheral tissue [19]. One recent study [14] suggested that activated DETC produce IL-17 during CHS and thus play a crucial role in CHS. The precise role of dermal γδ T cells in CHS, however, remains unclear.

In this study, we found that dermal γδ T cells are heterogeneous and have a distinct phenotype (CD44^hi^ CD62L^-^ CD103^+^ CD69^+^ E-lig^+^ P-lig^+^) reminiscent of αβ CD8^+^ T_RM_ in skin [20]. While we could readily demonstrate that dermal γδ T cells produce IL-17 and IL-22 upon activation, DETC did not. Interestingly, and consistent with their T_RM_-like phenotype, dermal γδ T cells showed an intermediate mode of skin residence, with the majority of cells remaining at their original site after 4 weeks of parabiosis, a rate of recirculation slower than dermal αβ T_MM_ cells but faster than authentic αβ CD8^+^ T_RM_, which showed no recirculation at this time point [20]. In agreement with one recently published study [14], we also found a significantly reduced primary CHS response in γδ T cell-deficient mice. Furthermore, using dermal γδ T cell-deficient chimeric mice, we demonstrated that dermal γδ T cells, and not DETC, were necessary for maximum CHS responses. We also showed that the reduced primary CHS response (as measured by ear swelling) was associated with loss of neutrophils skin infiltration during the challenge phase of CHS. Direct depletion of Gr-1^+^ neutrophils in vivo also significantly reduced ear swelling, suggesting that neutrophils are required for a full CHS response. Finally, through antibody-mediated depletion of Vγ4^+^ γδ T17 cells and through neutralization of IL-17 in vivo, we observed a significant reduction of the primary CHS response and impaired neutrophil recruitment. Overall, these results demonstrate that dermal γδ T17 cells contribute to the development of primary CHS, and that IL-17 production enhanced neutrophil infiltration, a major determinant of a robust CHS response.

## Materials and Methods

### Mice

CD45.1^+^ (002014), CD45.2^+^ (000664), GFP^+^ (003291), or TCR δ^-/-^ (002120) C57BL/6 mice were purchased from Jackson Lab and were bred and housed at the animal facility of the Harvard Institutes of Medicine. All experiments were performed under the protocols approved by the Harvard Medical School animal care and use committee. In general, 8–12 week-old mice were used for all experiments. CO_2_ was used to euthanize mice.

### Isolation and preparation of cells

Epidermis and dermis sheets were separated as previously described [21]. Briefly, ears were split into dorsal and ventral halves. The dermal sides were then floated down and incubated in 0.25% Trypsin and 2.5mM EDTA at 37°C for 30 min. Epidermis sheets were then gently peeled from the dermis. Both sheets were cut into small pieces and digested with 1 mg/ml Collagenase D (Rache) and 40 μg/ml DNase I (Roche) in Hanks balanced salt solution (HBSS) for 30–60 min. Enzymatic digestion was quenched by adding 10 mM EDTA and 2% FCS in HBSS. After filtering through a 70-μm nylon cell strainer, cells were collected and washed thoroughly with RPMI 1640 media before use.

### Parabiosis

Parabiotic surgery was performed as we have described previously [11, 20]. Briefly, sex- and age-matched GFP^+^ and GFP^−^ C57BL/6 mice were anaesthetized to full muscle relaxation with ketamine and xylazine (10 μg g^−1^) by i.p. injection. The corresponding lateral aspects of mice were shaved and the excess hair was wiped off with alcohol prep pad. After skin disinfection by wiping with betadine solution and 70% ethanol three times, two matching skin incisions were made from the olecranon to the knee joint of each mouse, and the subcutaneous fascia was bluntly dissected to create about 0.5 cm of free skin. The olecranon and knee joints were attached by a single 5–0 silk suture and tie, and the dorsal and ventral skins were approximated by staples or continuous suture. Betadine solution was used to cover the full length of the dorsal and ventral incision. The mice were then kept on heating pads and continuously monitored until recovery. 2.5 μg g^−1^ flunixin was used for analgesic treatment by subcutaneous injection every 8–12 h for 48 h after the operation. Mice were then monitored weekly with gel on the bottom of the cages. After an interval of the indicated weeks, parabiotic mice were surgically separated by a reversal of the above procedure for the next experiments.

### Chimeric mice

These mice were generated as described elsewhere [3,9]. Briefly, 8–10 x 10^6^ neonatal thymocytes (0–24 h after birth) were transferred intravenously to congenic C57BL/6 recipients that had been previously lethally irradiated with a split dose of 1300 rad (650 rad + 650 rad, 3 hours interval). One day later, 5 × 10^6^ congenic bone marrow (BM) cells from adult C57BL/6 mice were transferred. Chimeric mice were used at least 12 weeks later.

### DNFB-induced CHS model

The left ears of mice were sensitized epicutaneously with 0.25% DNFB for 2 consecutive days; 5 days later, the right ear was challenged with 0.25% DNFB. At 0–120 hours after challenge, the ear thickness was measured. In order to evaluate only the sensitization process, at 5 days after sensitization the left ear-draining lymph nodes (dLNs) and spleen were collected and the sensitized T and NK cells were analyzed. In some cases, mice were euthanized at 48 hours after challenge and their right ears were harvested for analysis. For transfer experiments, CD45.1^+^ naïve C57BL/6 mice were sensitized with 0.25% DNFB on both ears for 2 days; 5 days later, 2 x 10^7^ sensitized leukocytes isolated from dLNs were intravenously (i.v.) transferred to CD45.2^+^ recipient chimeric mice; 1 day later, these recipients were treated with DNFB on both ears. After an additional 48 hours, the ears were collected and analyzed.

### In vivo depletion of Vγ4^+^ cells, Gr-1^+^ neutrophils and neutralization of IL-17

Vγ4^+^ cell depletion was performed as described elsewhere [5]. Briefly, 400 μg anti-mouse Vγ4 antibody (Bio X Cell) or isotype antibody was injected intravenously into mice for 3 consecutive days. After the last injection, these mice were treated with DNFB to induce acute CHS. For depletion of Gr-1^+^ neutrophils or neutralization of IL-17, mice were sensitized and treated i.p. with 100 μg anti-Gr-1 (eBioscience) or 100 μg anti-IL-17 (BioLegend) 1 day prior to DNFB challenge.

### Intracellular cytokine detection

The skin cells were prepared as described above. Lymphocytes from LNs and splenocytes were prepared as usual. Red blood cells were lysed using erythrocyte lysis buffer. Cells were then incubated with 50 ng/ml PMA and 1 mM Ionomycin at the presence of Brefeldin A for 6–7 hours. In some cases, skin cells were incubated with 0.25% DNFB instead of PMA / Ionomycin. Fc receptors were blocked with CD16/CD32 mAbs and intracellular IL-17, IL-22, IFN-γ, IL-4 and TNF-α staining was performed using Intracellular Cytokine Detection Kits (BD Bioscience) before flow cytometry.

### Antibodies and flow cytometry analysis

The following anti-mouse antibodies were obtained from BD PharMingen: TCR γδ (GL3), TCR β (H57-597), TCR Vγ5 (536), TCR Vγ4 (UC3-10A6), CD62L (MEL-14), CD44 (IM7), CD8α (53–6.7), CD69 (H1.2F3), CD103 (M290), IL-17 (TC11-18H10), IFN-γ (XMG1.2), IL-4 (11B11), TNF- α (MP6-XT22), CD3 (145-2C11), CD4 (L3T4), NK1.1 (PK136), CD45.1 (A20), CD11b (M1/70), CD16/CD32 (2.4G2), Gr-1 (RB6-8C5). Rat IgG2b (eB149/10H5) was purchased from eBioscience) and rat IgG1 (RTK2071) was obtained from BioLegend. Anti-mouse IL-22 (Clone 140301) was purchased from R&D systems. E- or P-selectin ligand (E- or P-lig) expression was examined by incubating cells with rmE-Selectin/Fc Chimera or rmP-Selectin/Fc Chimera (R&D Systems) in conjunction with APC-conjugated F(ab')_2_ fragments of goat anti-human IgG F(c) antibody (Jackson Immunoresearch). Dead cells were excluded using 7-AAD staining. Data were analyzed on FACSCanto^™^ Flow Cytometer using FACSDiva software.

### H&E staining

To examine the histology of DNFB-challenged skin tissues, samples were collected 48 hours after DNFB challenge and fixed in 10% neutral buffered formalin solution. Paraffin-embedded tissue sections were made and stained with H&E by a rodent histopathology core facility at Harvard Medical School.

### Statistical analysis

Statistical significance in values between experimental groups was determined by one-way analysis of variance (ANOVA) followed by Tukey post-test. P<0.05 was considered statistically significant.

## Results

### Dermal γδ T cells have distinct TCR repertoire, express CD103^+^CD69^+^, and produce IL-17 and IL-22 upon activation

When we digested total skin samples of post natal mice, two discrete γδ T cell populations were observed: one expressed an intermediate level of γδ TCR (γδ TCR^inter^) while the other was γδ TCR^hi^. After separating epidermis and dermis, we found that γδ TCR^inter^ cells were located in dermis while γδ TCR^hi^ cells are epidermal DETC. DETC are Vγ5^+^ but γδ T cells in dermis are heterogeneous and include a significant Vγ4^+^ population (S1A Fig). These findings are consistent with recently published reports [3,4]. Dermal γδ T cells have T effector memory phenotype (CD44^hi^CD62L^–^) and express the skin homing molecules --- E- and P-lig. Our previous studies had demonstrated that embryonic trafficking of DETC to skin is dependent on E-lig and CCR4 [21]. Interestingly, the majority of dermal γδ T cells also express CD69 and CD103 (S1B Fig), two molecules typically expressed by tissue resident memory T cells (T_RM_) [20, 22–24]. In order to evaluate the cytokine productions of γδ T cells in the skin, we used PMA and ionomycin to stimulate the skin cells prepared freshly from intact naive skin. As shown in S1C Fig, only dermal γδ T cells (and not DETC) release large amounts of IL-17 and IL-22; while IFN-γ, IL-4 and TNF-α are not produced by either population of γδ T cells in the skin. Taken together, these data suggest that dermal γδ T cells have phenotypic and functional features that are quite distinct from DETC.

### Dermal γδ T cells show a novel intermediate pattern of tissues residence

**Fig 1:**
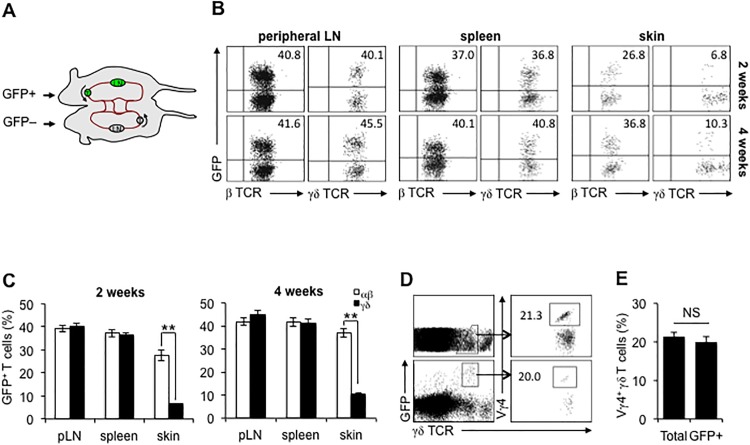
The circulation of dermal γδ T cells is limited at steady state. A, parabiotic mice were created by surgically joining the flank and tying the limbs of age- and sex-matched GFP^+^ and GFP^−^ mice. B and C, at 2 or 4 weeks after surgery, peripheral LNs (pLN), spleen, and skin were collected from GFP^−^ parabiotic mice and the frequencies of GFP^+^ αβ or γδ T cells were analyzed. D and E, at 4 weeks after surgery, the skin of GFP^−^ parabiotic mice was harvested and the frequency of Vγ4^+^ cells in total or GFP^+^ dermal γδ T cells was determined. The number in the quadrant represents the percentage. **: P<0.01. NS: no significant. FACS results are representative of three independent parabiosis experiments (4 parabiotic pairs for each time point).

Previously published intravital imaging data showed that dermal γδ T cells are mobile within the skin [3,4]; however, their ability to leave the skin and enter circulation has not been assessed. In order to explore their capacity to exit skin and circulate into blood, we generated GFP^+^:GFP^−^ parabiotic mice (Fig 1A). Our previous data showed that a shared circulatory system between two parabionts can be established at 2 weeks after surgery; and by 4 weeks, freely circulating lymphocytes approach equilibrium in secondary lymphoid organs (SLOs) of each parabiont [20]. We thus separated the parabiotic mice at 2 or 4 weeks after surgery and analyzed circulating GFP^+^ αβ or γδ T cells in SLOs and skin of GFP^−^ parabiont. As shown in Fig 1B and 1C, the percentages of GFP^+^ T cells from 2 weeks to 4 weeks in either peripheral LN (αβ T cells: 40.8%→41.76%; γδ T cells: 40.1%→45.5%) or spleen (αβ T cell: 37%→40.1%; γδ T cells: 36.8%→40.8%) were gradually increased and approaching 50%, consisting with our and other's reports [20, 25] that T cells in SLOs of parabiotic mice circulate freely and reach equilibrium quickly. However, we found that the percentage of GFP^+^ αβ T cells in the skin at 2 or 4 weeks after surgery was 26.8% and 36.8%, respectively, significantly lower than those in SLOs, consistent with the recently described population of αβ TCR T_MM_ [26, 27]. More surprisingly, the frequency of GFP^+^ γδ T cells in the non-GPF mouse skin was only 6.8% and 10.3% at 2 and 4 weeks after surgery, respectively. No GFP^+^ DETC were detected. In our studies with αβ TCR T_RM_ induced by skin immunization, these cells did not re-circulate measurably. These results indicate that T cells in skin have multiple patterns of tissue residence and migratory capacity: non-re-circulating DETC and αβ TCR T_RM_ [11, 20], very slowly re-circulating cells (dermal γδ T cells), and more rapidly re-circulating cells (TCR αβ T_MM_). Considering the heterogeneity of TCR repertoire in dermal γδ T cells (S1 Fig), we further analyzed the TCR repertoire of circulating GFP^+^ dermal γδ T cells and found that the proportion of Vγ4^+^ cells was 20%, which was quite similar to the proportion of Vγ4^+^ cells in total dermal γδ T cells (21.3%) (Fig 1D and 1E). Although we were not able to determine the proportions of other γδ TCR^+^ populations in dermis due to the unavailability of specific antibodies, the above data suggest that slowly re-circulating dermal γδ T cells represents a diverse population.

### The acute skin CHS response is significantly reduced in dermal γδ T cell-deficient chimeric mice

**Fig 2:**
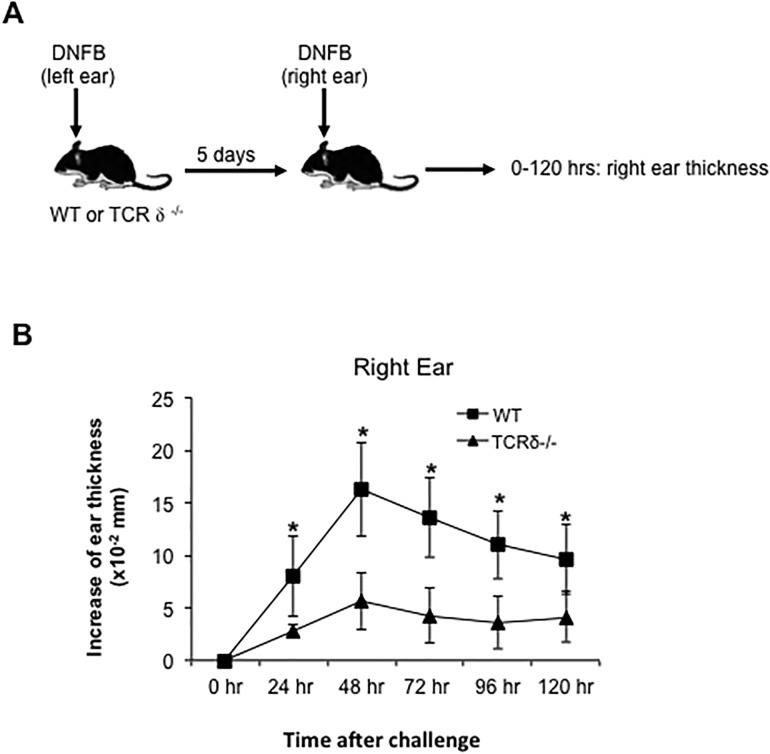
CHS response is remarkably reduced in TCR δ^-/-^ mice. WT or TCR δ^-/-^ mice were sensitized with 0.25% DNFB on left ears for 2 consecutive days. 5 days later, the right ears were challenged with one dose of 0.25% DNFB. The ear thickness was measured at 0–120 hours after challenge (A and B). *: P<0.05. 5 mice for each group were tested. Results are representative of two independent experiments.

**Fig 3:**
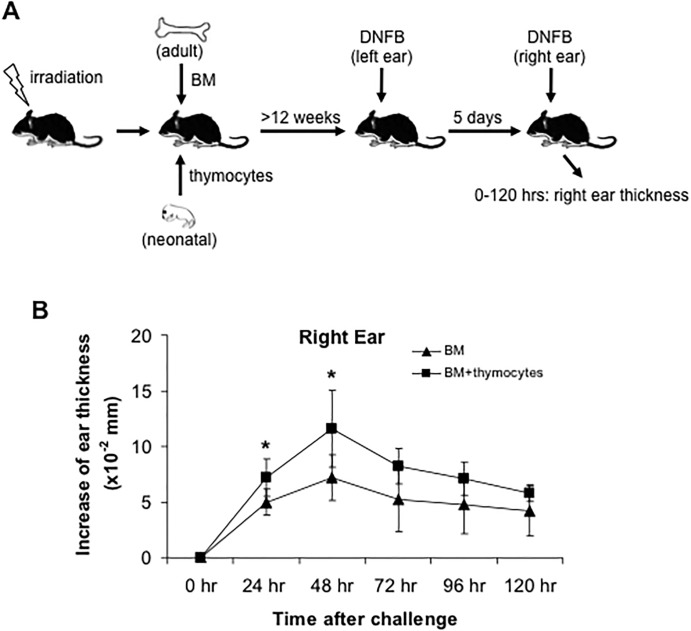
CHS response is significantly reduced in dermal γδ T cell-deficient chimeric mice. A, Generation of chimeric mice. 5–10 x 10^6^ neonatal thymocytes from newborn C57BL/6 mice (day 0–1 after birth) were transferred to half of the irradiated mice. 24 hours later, 5 x 10^6^ bone marrow (BM) cells from adult naïve C57BL/6 mice were intravenously transferred to all irradiated mice. At least 12 weeks later, these BM or BM + thymocytes chimeric mice were sensitized with 0.25% DNFB on left ears at first two days. 5 days later, the right ears were challenged with one dose of 0.25% DNFB. The ear thickness was measured at 0–120 hours after challenge (B). *: P<0.05. 5 mice for each group were tested. Results are representative of two independent experiments.

Recently dermal γδ T cells were implicated in the pathogenesis of murine models of psoraisis [6–9]. However, whether these T cells play an important role in other inflammatory skin diseases such as acute CHS is not clear. In order to address this question, we first sensitized and challenged TCR δ^-/-^ mice with 0.25% DNFB (Fig 2A). Compared to wild type (WT) mice, γδ T cell-deficient mice showed a markedly reduced primary CHS response (Fig 2B), suggesting that γδ T cells are important mediator in early CHS. Certain studies have suggested that DETC can respond to contact allergen, but their exact role in CHS is still debated [15]. In order to determine the role of dermal γδ T cells in CHS at the presence of DETC, we next generated only dermal γδ T cell-deficient chimeric mice as previously described [3,9]. After irradiation, recipients of bone marrow (BM) cells alone have few if any dermal γδ T cells (though they have normal DETC), while the mice with both BM and neonatal thymocyte transfers have a normal complement of dermal γδ T cells. DETC are equivalent in both mice since they are radioresistant. We sensitized these chimeric mice with 0.25% DNFB on the left ears for 2 consecutive days. After 5 days, the right (un-sensitized) ears were challenged with one dose of 0.25% DNFB and their thickness was measured at 24 hour intervals (Fig 3A). Compared to the controls, dermal γδ T cell-deficient chimeric mice showed a significant reduction of acute CHS response, which peaked at 48 hours (P = 0.04) after DNFB challenge (Fig 3B). Therefore, these data suggest that dermal γδ T cells are involved in the acute CHS response, and that DETC play no measurable role.

### Hapten specific αβ T or NK cell sensitization is normal in dermal γδ T cell-deficient chimeric mice

Most published studies suggest that the acute CHS response is mediated by CD8^+^ T cells, CD4^+^ T cells, and NK cells [18, 28]. In an acute CHS model, the antigen specific effector cells are generated during sensitization phase and migrate to skin, and during the challenge phase these effectors cells are activated induce skin inflammation. To determine the possible influence of dermal γδ T cells on the generation of effector cells during sensitization phase, dermal γδ T cell-deficient chimeric mice were sensitized with 0.25% DNFB for 2 consecutive days. After 5 days, dLNs and spleen were harvested for analysis of effector cells (S2 Fig). Our results indicated no difference between control mice and dermal γδ T cell-deficient chimeric mice with regard to CD8^+^ and CD4^+^ T cells or NK cells in dLNs and spleen (S2A, S2B, S2D and S2E Fig). IFN-γ and IL-17 have been considered as two crucial inflammatory cytokines that are required for optimal CHS response [17, 29]. However, in the present study, these two cytokines produced either by CD8^+^, CD4^+^, or by NK cells in dLNs or spleen were comparable in dermal γδ T cell-deficient and sufficient chimeric mice (S2C and S2F Fig). Taken together, these results indicate that the absence of dermal γδ T cells does not impair the sensitization of hapten specific αβ T or NK cells.

### The recruitment of Gr-1^+^CD11b^+^ neutrophils to skin is remarkably reduced in dermal γδ T cell-deficient chimeric mice during challenge phase

**Fig 4:**
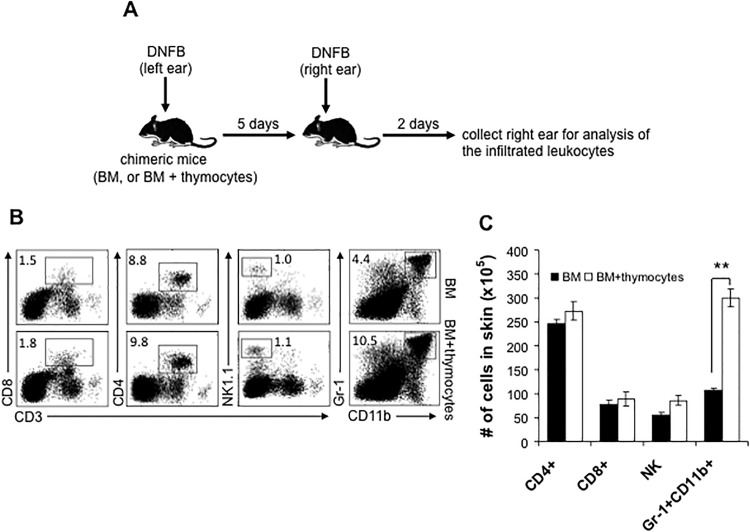
The recruitment of Gr-1^+^ CD11b^+^ neutrophils, not the sensitized CD4^+^ or CD8^+^ T cells and NK cells, to DNFB-challenged skin is significantly influenced in dermal γδ T cell-deficient chimeric mice. A, The DNFB-induced CHS model using chimeric mice was established as described in Fig 3. 48 hours after DNFB challenge, right ears were collected and digested to prepare single cell suspensions. After washed with cold PBS, skin cells were stained with fluorescence-conjugated antibodies for flow cytometry. B, The frequencies of CD4^+^ or CD8^+^ T cells, NK cells, and Gr-1^+^ CD11b^+^ neutrophils in the challenged skin. C, The numbers of skin infiltrated CD4^+^ or CD8^+^ T cells, NK cells, and Gr-1^+^ CD11b^+^ neutrophils. **: P<0.01. Results are representative of two independent experiments.

**Fig 5:**
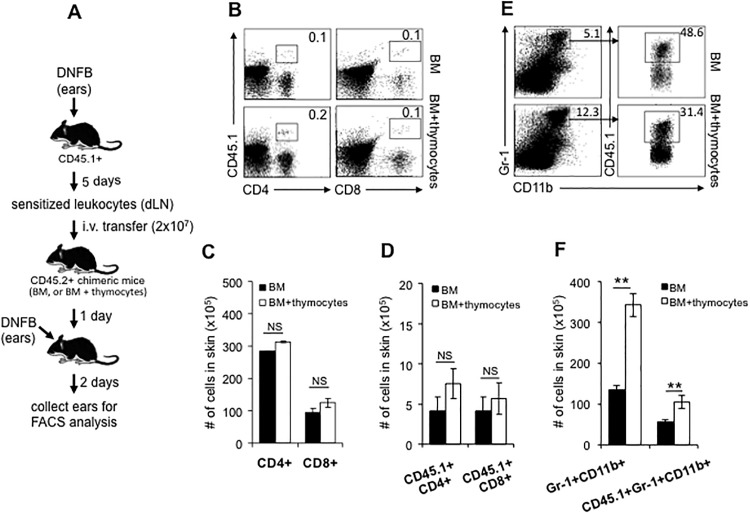
The sensitized CD4^+^ or CD8^+^ T cells infiltrate into DNFB-treated skin normally after intravenous transfer while Gr-1^+^ CD11b^+^ neutrophils are greatly reduced in dermal γδ T cell-deficient chimeric mice. A, Wild type CD45.1^+^ C57BL/6 mice were sensitized with DNFB at first 2 days. 5 days later, dLNs were collected to prepare cell suspension. After washed with PBS, 2 x 10^7^ CD45.1^+^ sensitized leukocytes were intravenously transferred to CD45.2^+^ BM or BM+thymocytes chimeric mice. 1 day after transfer, the ears of chimeric mice were treated with 0.25% DNFB. 48 hours later, the treated ears were collected for flow cytometry analysis. B, The frequency of total or donor T cells. C and D are the numbers of total or donor T (CD4^+^ or CD8^+^) cells in the skin, respectively. E and F are the frequency and number of total or donor Gr-1^+^CD11b^+^ neutrophils in the skin, respectively. NS: no significant. **: P<0.01. Results are representative of two independent experiments.

**Fig 6:**
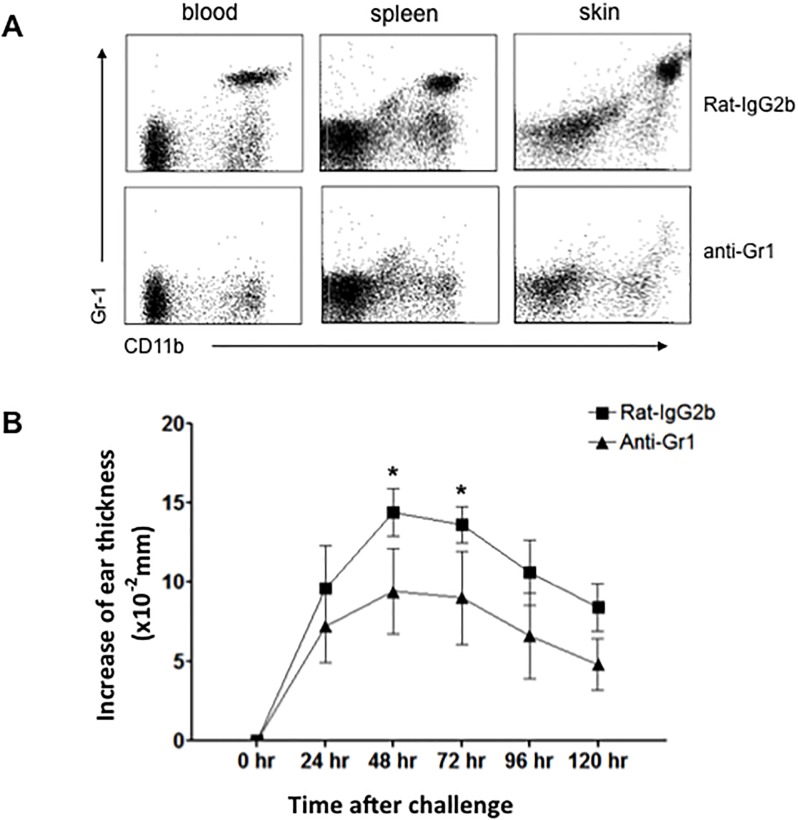
CHS response is reduced significantly after depletion of Gr-1^+^ neutrophils in vivo. Naive C57BL/6 mice were treated with DNFB as Fig 2 to generate DNFB-CHS model. 1 day prior to DNFB challenge, mice were i.p. injected with 100 μg anti-Gr-1 or 100 μg isotype antibodies (rat-IgG2b). Neutrophils (CD11b^+^ Gr-1^+^) in blood, spleen, and challenged skin site at 48 hours after DNFB challenge were analyzed by FACS (A). The ear thickness was measured at 0–120 hours after challenge (B). *: P<0.05. 5 mice for each group were tested. Results are representative of three independent experiments.

**Fig 7:**
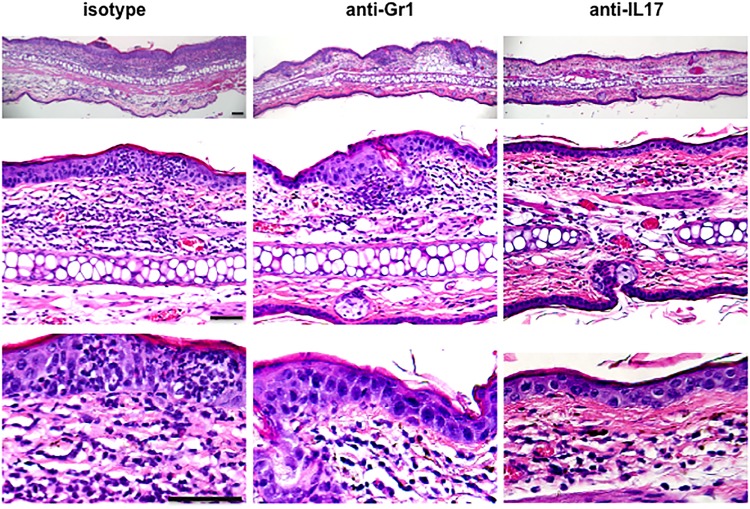
H&E staining of DNFB-challenged skin. Naive C57BL/6 mice were treated with DNFB as Fig 2 to generate DNFB-CHS model. 1 day prior to DNFB challenge, mice were i.p. injected with 100 μg anti-Gr-1, 100 μg anti-IL-17, or 100 μg isotype antibodies. 48 hours after DNFB challenge, the challenged skin site was harvested for H&E staining. 5 mice for each group were tested. Results are representative of two independent experiments. The bar represents 100 μm.

We next asked if the recruitment of αβ T or NK effector cells to the challenged skin was influenced by the absence of dermal γδ T cells. To address this question, dermal γδ T cell-deficient chimeric mice were sensitized and challenged with DNFB as described before. At 48 hours after challenge, skin tissue was collected and leukocyte infiltration was analyzed (Fig 4A). Interestingly, the results showed that the frequencies and numbers of CD8^+^ or CD4^+^ T cells and NK cells in the skin were neither reduced nor increased in dermal γδ T cell-deficient chimeric mice (Fig 4B and 4C), suggesting that the absence of dermal γδ T cells does not significantly influence the skin infiltration of these effector cells after hapten challenge. There was, however, a striking reduction of Gr-1^+^CD11b^+^ neutrophils in the challenged skin of dermal γδ T cell-deficient chimeric mice (Fig 4B and 4C). We then sensitized the skin of naïve CD45.1^+^ C57BL/6 mice with 0.25% DNFB. 5 days later, we adoptively transferred 2 × 10^7^ CD45.1^+^ cells that were freshly prepared from dLNs at day 5 into naïve CD45.2^+^ dermal γδ T cell-deficient or control chimeric mice. After 24 hours, 0.25% DNFB was topically applied to these mice. At 48 hours after DNFB application, we harvested DNFB-treated skin to analyze the infiltration of effector cells (Fig 5A). As shown in Fig 5B, 5C and 5D, the skin infiltrations of either total or CD45.1^+^ donor-derived CD4^+^ or CD8^+^ T cells were comparable between dermal γδ T cell-deficient chimeric mice and control mice. The skin infiltration of CD45.1^+^ donor-derived T cells (either CD8^+^ or CD4^+^) showed a downward trend but it was not statistically significant. Again, however, a marked reduction of either total or CD45.1^+^ donor-derived Gr-1^+^CD11b^+^ neutrophils was detected in DNFB-treated skin (Fig 5E and 5F). The neutrophil infiltration to the skin has previously been reported as characteristic of acute murine contact hypersensitivity [17,18, 29]. In order to evaluate the effect of this reduction of neutrophil infiltration on CHS response, we depleted Gr-1^+^ neutrophils in vivo by i.p. injection of anti-Gr-1 antibody. This resulted in a significantly reduced CHS response (Fig 6A and 6B) and less skin inflammation and cell infiltration (Fig 7) were found. Therefore, the results shown here demonstrate that dermal γδ T cells have primary effects in regulating neutrophil infiltration during challenge phase of CHS response.

### IL-17-producing dermal γδ T cells increase in number in situ after DNFB challenge and contribute to CHS response

**Fig 8:**
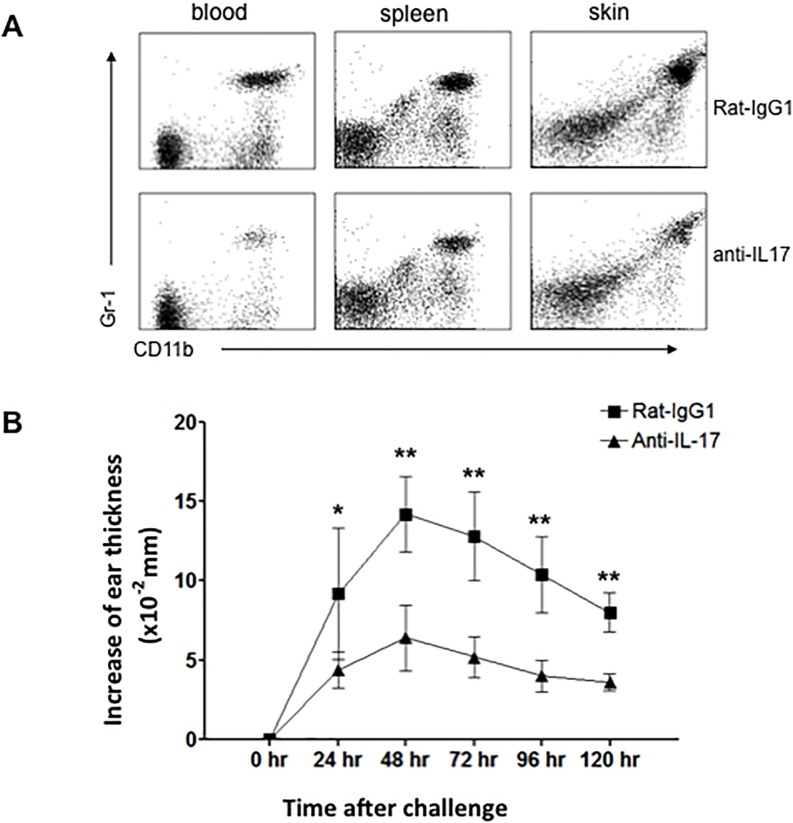
CHS response is dramatically reduced after neutralization of IL-17 in vivo. Naive C57BL/6 mice were treated with DNFB as Fig 2 to generate DNFB-CHS model. 1 day prior to DNFB challenge, mice were i.p. injected with 100 μg anti-IL-17 or 100 μg isotype antibodies (rat-IgG1). Neutrophils (CD11b^+^ Gr-1^+^) in blood, spleen, and challenged skin site at 48 hours after DNFB challenge were analyzed by FACS (A). The ear thickness was measured at 0–120 hours after challenge (B). **: P<0.01. 5 mice for each group were tested. Results are representative of three independent experiments.

**Fig 9:**
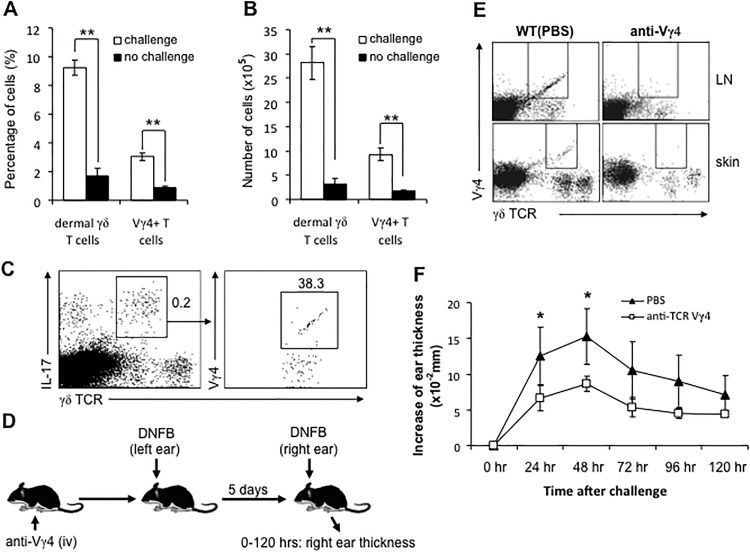
Dermal γδ T cells rapidly increase in situ and produce large amounts of IL-17 after DNFB challenge. The DNFB-induced CHS model using wild type C57BL/6 mice was established as described in Fig 2. 24 hours after DNFB challenge, right ears were collected and the frequency and number of dermal γδ T cells were analyzed by flow cytometry (A: of total skin cells; and B: per ear). In parallel, parts of skin cells were incubated in RPMI 1640 media supplemented with 5% FCS, 1% P/S, 0.25% DNFB and Brefeldin A at 37°C. 6–7 hours later, IL-17 production of total dermal γδ T cells and the proportion of IL-17-producing Vγ4^+^ cells were analyzed by intracellular staining (C). D, Vγ4^+^ γδ T cells were depleted by i.v. injection of anti-Vγ4 antibody or isotype antibody for 3 consecutive days. DNFB-induced CHS model was created as described in Fig 2. Ear swelling was measured during 0–120 hours after challenge. E: Vγ4^+^ cells were depleted in LN and skin after 3 consecutive days of anti-Vγ4 injection. F: The increase of ear thickness after DNFB challenge. *: P<0.05. **: P<0.01. 5 mice for each group were tested. Results are representative of three independent experiments.

In order to assess the contribution of IL-17 to ear swelling and Gr-1+ neutrophil accumulation, we treated mice in vivo with a neutralizing antibody to IL-17 (Fig 8A). This resulted in a dramatically reduced CHS response (Fig 8B), less skin inflammation and neutrophil infiltration (Figs 7 and 8A). In order to explore how dermal γδ T cells regulate neutrophil infiltration after DNFB challenge, we used naïve C57BL/6 mice to generate DNFB-CHS as described above. At 24 hours after DNFB challenge, we found that dermal γδ T cells have significantly increased in number at challenged site (Fig 9A and 9B). Notably, in DNFB-challenged site Vγ4^+^ T cell population was increased almost 5 times (Fig 9A and 9B). It was reported that Scart1^+^ γδ TCR^+^ cells infiltrate both the dermis and epidermis in 12-O-tetradecanoyl-phorbol-13-acetate (TPA)-induced skin inflammation model [30]. Our data do not allow us to attribute this increase in γδ T cells to recruitment from blood, local proliferation, or local chemotactic accumulation from surrounding dermis, respectively. We did not detect any significant changes in DETC after challenge (data not shown). Given that dermal γδ T cells produce IL-17 upon activation and IL-17 is involved in regulating neutrophil infiltration during acute inflammation [31], we asked whether dermal γδ T cells could regulate neutrophil infiltration by producing early IL-17 during challenge phase of CHS response. To explore this, we first incubated the skin cells (freshly prepared from DNFB-challenged skin of C57BL/6 mice at 24 hours after challenge) with 0.25% DNFB in the media ex vivo for 6–7 hours. IL-17 production was measured by intracellular staining. As shown in Fig 9C, almost half of dermal γδ T cells were positive for IL-17 (0.2% of total skin cells), and 38.3% were Vγ4^+^. Due to the antibody unavailability in this study we were not able to test Vγ6^+^ T17 cells that have been demonstrated to be another important γδ T17 population [32,33].

We next injected C57BL/6 mice with anti-Vγ4 antibodies intravenously for 3 consecutive days to deplete Vγ4^+^ cells in vivo, as described elsewhere [5]. The mice were then sensitized and challenged with 0.25% DNFB to induce CHS response (Fig 9D). Fig 9E shows the depletion of Vγ4+ cells in LN and skin. The results showed that CHS ear swelling was significantly reduced in Vγ4-depleted mice after DNFB challenge, compared to that of wild type control mice (Fig 9F).

Taken together, our results suggest that dermal γδ T cells are rapidly increased in situ after DNFB challenge, produce more IL-17, and thus promote abundant neutrophils infiltrate into skin during challenge phase of CHS.

## Discussion

In this study we characterized the phenotype, migratory behavior, and cytokine production of γδ T cells in murine skin. Our results showed that both newly identified dermal γδ T cells and DETC are CD44^hi^CD62L^–^, and share certain tissue retention markers with αβ TCR T_RM_ (CD103+, CD69^+^). We also found that only dermal γδ T cells, and not DETC, produce IL-17 and IL-22 upon activation in vitro. Importantly, our parabiosis experiments revealed that in unperturbed skin, dermal γδ T cells exit skin very slowly as compared to dermal αβ T cells in circulating to bloodstream (DETC do not circulate at all). While αβ T cells elicited by an infection or antigen challenge become T_RM_ and do not recirculate [11,20], under normal conditions it has been shown in mice and humans that a subpopulation of T cells in skin are migratory (hence migratory memory T cells, or T_MM_) [26,27]. We next demonstrated γδ T cells are important effector cells in acute CHS using TCR δ^-/-^ mice. We then generated chimeric mice deficient only in dermal γδ T cells and found a significant reduction of acute CHS response in these mice. To our knowledge, this is the first report on the effect of dermal γδ T cells in the development of acute CHS. Furthermore, we demonstrated that the reduced CHS response, as determined by ear swelling (i.e., inflammatory edema), was related to impaired skin-infiltration of Gr-1^+^CD11b^+^ neutrophils during the challenge phase of CHS, which was in turn related to decreased IL-17 production. Finally, we found that these slowly circulatory dermal γδ T cells rapidly increased in situ after DNFB challenge and produced more IL-17; depletion of IL-17-producing Vγ4^+^ dermal γδ T cells or neutralization of IL-17 led to a significantly reduced CHS response and diminished neutrophil infiltration. These data suggest that dermal γδ T cell-derived IL-17 plays an important role in acute CHS. It is important to reinforce that murine CHS models measure a primary immune response to contact sensitizers, and in this setting γδ T cells are clearly important. A recent study [11] showed that long-term memory to CHS was mediated by αβ T cells, though the contribution of γδ T cells was not specifically measured.

γδ T cells were identified in murine dermis some time ago [3, 4]. Like DETC [21], we show that dermal γδ T cells are also CD44^hi^ and CD62L^–^, and they express the skin-homing addressins E- and P-lig. Consistent with the published reports [3,4,5], γδ T cells in dermis are heterogeneous in γδ TCR repertoires and produce IL-17 and IL-22 upon activation. Our data in S1 Fig and Fig 1 showed some difference in the fraction of Vγ4+ cells. This might be related to the age of the mice used since it was reported that the frequency of dermal Vγ4+ cells varies with the age of the animal [32]. It has been demonstrated that in dermis more than 50% IL-17 is derived from Vγ4^+^ cells, which constitutively express CCR6, RORγt, and IL-23R [5]. In addition, it is noteworthy that both γδ T cells at epidermis (DETC) and dermis express CD69 and CD103. Emerging evidence [20, 22–24, 34,35] recently suggests that these two molecules may contribute to the retention of memory T cells in the peripheral tissues. One recent study [4] using intravital microscopy showed that DETC are stationary, while some dermal γδ T cells move at a low speed (mean velocity <5 μm/min) within the skin and others remain sessile. Considering the epidermal localization of stationary DETC and skin-resident memory CD8^+^ T (T_RM_) cells [20, 22] and identification of circulating memory CD4+ T cells in dermis [26], we assume that these slowly circulatory γδ T cells might mainly localize in a special site in dermis.

Given the distinct characteristics of dermal γδ T cells, their potential roles in skin inflammatory diseases have been proposed when they were identified [3,4]. In recent years, emerging evidence from both human and mouse skin studies [5–9] has suggested that dermal γδ T17 cells are critical immune cells in the development of psoriasisiform dermatitis. In this study we first used TCR δ^-/-^ mice and demonstrated a key role of γδ T cells in CHS. Although one recent study[14] using TCR δ^-/-^ mice also reported a reduced CHS response, there are completely different findings existing in the literature about the role of different γδ T cell populations in CHS [12,13,15]. Different protocols, chemical reagents, and different mouse genetic background may cause this discrepancy. Since TCR δ^-/-^ mice lack of all γδ T cells, it is hard to know if skin-resident γδ T cells or γδ T cells in SLOs mediate the reduced CHS response. Two independent laboratories [3,4] found that DETC are radioresistant and cannot be replenished from the bone marrow in adult mice, while dermal γδ T cells fail to reconstitute following irradiation. In concordance with these groups, we generated dermal γδ T cell-deficient chimeric mice, which contains few if any dermal γδ T cells but have normal DETC. Therefore, this is a powerful model to address the relative role of dermal γδ T cells in CHS. We found that primary CHS response in these chimeric mice lacking dermal γδ T cells is significantly reduced. It is generally accepted that the activated CD8^+^ T cells play an important role in both acute and chronic CHS [18]. Our data suggests that dermal γδ T cells are dispensable for the sensitization of hapten-specific T cells. Nevertheless, a greatly impaired infiltration of Gr-1^+^CD11b^+^ neutrophils was observed in dermal γδ T cell-deficient chimeric mice after acute hapten challenge. In agreement with a recent report [36], we depleted Gr-1^+^ neutrophils in vivo and also found a significantly reduced CHS response. Therefore, we believe that dermal γδ T cells may participate in early infiltration of inflammatory cells (such as neutrophils), therefore contributing to CHS that peaks at 48 hours after hapten challenge.

Neutrophil infiltration is a hallmark of acute inflammation and is regulated by IL-17 through the production of neutrophil tropic and chemotactic chemokines [31]. In IL-17-deficient mice, a marked suppression of the CHS response and decreased infiltration of neutrophils were reported but the early cellular source of IL-17 was not revealed [17]. In this study we found that not only total dermal γδ T cells but also dermal γδ T17 cells (including Vγ4^+^ γδ T17 cells) were greatly and rapidly increased in challenged skin site, suggesting that dermal γδ T cells respond very quickly after DNFB challenge. Through in vivo depletion of Vγ4^+^ γδ T17 cells and neutralization of IL-17, we have demonstrated that dermal γδ T cell-derived IL-17 is an important early cytokine involved in regulating neutrophil infiltration during challenge phase of CHS response. In addition, γδ T cells can be rapidly activated to produce IL-17 merely through exposure to some cytokines, such as IL-23 and IL-1β [37]. Therefore, it will be interesting to determine whether dermal DC, macrophages, or other cells can rapidly secret these cytokines to activate and promote expansion of dermal γδ T cells through cytokine-mediated signaling after DNFB challenge.

In summary, our studies have demonstrated that dermal γδ T cells show a unique profile of tissue residence and slow re-circulating, but can rapidly increase in number in situ and produce large amounts of IL-17 after DNFB challenge. The consequence of this is to promote neutrophil infiltration during CHS. These findings help clarify our understanding of dermal γδ T cells in pathogenesis of the skin inflammatory diseases and also provide information for the development of new therapeutic strategies.

## Supporting Information

S1 Fig Dermal γδ T cells have a unique characteristic. The ears of normal naive C57BL/6 mice at the age of 8–12 weeks were harvested and separated into dorsal and ventral sheets. Sheets were then chopped into small pieces and digested with RPMI 1640 supplemented with 5% FCS, 1% P/S, 1 mg/ml Collegenase D and 40 μg/ml DNase I at 37°C for 1 hour. In some cases, dorsal sheets were floated dermis side down on a 5% dispase solution for 20–30 min. Epidermis and dermis were gently separated and chopped into small pieces for digestion. Digested skin tissues were then mashed through a 70-μm nylon cell strainer to collect cell suspensions. After washed thoroughly with cold PBS, cells were stained with fluorescence-conjugated antibodies (A and B). E- or P-selectin ligands (E- or P-lig) were detected by CD62E/Fc or CD62P/Fc chimera in a calcium-dependent binding manner, respectively. C, skin cells were incubated with DMEM (10%FCS) containing 50 ng/ml PMA and 1 mM Ionomycin in the presence of Brefeldin A at 37°C for 6 hours. Cytokine productions of skin γδ T cells were measured by intracellular staining. Results are representative of at least three independent experiments.
(TIFF)

S2 Fig The sensitization of CD4^+^ or CD8^+^ T cells and NK cells is normal in dermal γδ T cell-deficient chimeric mice. The ears of chimeric mice were sensitized with 0.25% DNFB for 2 consecutive days. 5 days later, draining lymph node (dLN) and spleen were harvested and CD4^+^ or CD8^+^ T cells and NK cells as well as their IL-17 / IFN-γ productions (measured as described at Fig 1) were analyzed by flow cytometry. dLN: A (percentage), B (cell numbers), and C (cytokine productions); spleen: D (percentage), E (cell numbers), and F (cytokine productions). Results are representative of two independent experiments.
(TIFF)

## References

[1] Nanno M, Shiohara T, Yamamoto H, Kawakami K, Ishikawa H. gammadelta T cells: Firefighters or fire boosters in the front lines of inflammatory responses. Immunol Rev. 2007;215:103–113. 17291282 10.1111/j.1600-065X.2006.00474.x

[2] Macleod AS, Havran WL. Functions of skin-resident γδ T cells. Cell Mol Life Sci. 2011;68:2399–2408. 10.1007/s00018-011-0702-x 21560071 PMC3123394

[3] Gray EE, Suzuki K, Cyster JG. Identification of a motile IL-17-producing gammadelta T cell population in the dermis. J Immunol. 2011;186:6091–6095. 10.4049/jimmunol.1100427 21536803 PMC3098921

[4] Sumaria N, Roediger B, Ng LG, Qin J, Pinto R, Cavanagh LL, et al Cutaneous immunosurveillance by self-renewing dermal gammadelta T cells. J Exp Med. 2011;208:505–518. 10.1084/jem.20101824 21339323 PMC3058585

[5] Cai Y, Shen X, Ding C, Qi C, Li K, Li X, et al Pivotal role of dermal IL-17-producing γδ T cells in skin inflammation. Immunity. 2011;35:596–610. 10.1016/j.immuni.2011.08.001 21982596 PMC3205267

[6] Mabuchi T, Takekoshi T, Hwang ST. Epidermal CCR6+ γδ T cells are major producers of IL-22 and IL-17 in a murine model of psoriasiform dermatitis. J Immunol. 2011;187:5026–5031. 10.4049/jimmunol.1101817 21984702

[7] Pantelyushin S, Haak S, Ingold B, Kulig P, Heppner FL, Navarini AA, et al Rorγt+ innate lymphocytes and γδ T cells initiate psoriasiform plaque formation in mice. J Clin Invest. 2012;122:2252–2256. 10.1172/JCI61862 22546855 PMC3366412

[8] Becher B, Pantelyushin S. Hiding under the skin: Interleukin-17-producing γδ T cells go under the skin? Nat Med. 2012;18:1748–1750. 10.1038/nm.3016 23223063

[9] Gray EE, Ramírez-Valle F, Xu Y, Wu S, Wu Z, Karjalainen KE, et al Deficiency in IL-17-committed Vγ4(+) γδ T cells in a spontaneous Sox13-mutant CD45.1(+) congenic mouse substrain provides protection from dermatitis. Nat Immunol. 2013;14:584–592. 10.1038/ni.2585 23624556 PMC3660499

[10] Peiser M, Tralau T, Heidler J, Api AM, Arts JH, Basketter DA, et al Allergic contact dermatitis: epidemiology, molecular mechanisms, in vitro methods and regulatory aspects. Cell Mol Life Sci. 2012;69:763–781. 10.1007/s00018-011-0846-8 21997384 PMC3276771

[11] Gaide O, Emerson RO, Jiang X, Gulati N, Nizza S, Desmarais C, et al Common clonal origin of central and resident memory T cells following skin immunization. Nat Med. 2015; 21:647–653. 10.1038/nm.3860 25962122 PMC4632197

[12] Girardi M, Lewis J, Glusac E, Filler RB, Geng L, Hayday AC, et al Resident skin-specific gammadelta T cells provide local, nonredundant regulation of cutaneous inflammation. J Exp Med. 2002;195:855–867. 10.1084/jem.20012000 11927630 PMC2193718

[13] Guan H, Zu G, Slater M, Elmets C, Xu H. GammadeltaT cells regulate the development of hapten-specific CD8+ effector T cells in contact hypersensitivity responses. J Invest Dermatol. 2002;119:137–142. 10.1046/j.1523-1747.2002.01830.x 12164936

[14] Nielsen MM, Lovato P, MacLeod AS, Witherden DA, Skov L, Dyring-Andersen B, et al IL-1β-dependent activation of dendritic epidermal T cells in contact hypersensitivity. J Immunol. 2014;192:2975–2983. 10.4049/jimmunol.1301689 24600030 PMC4020443

[15] Kaplan DH, Igyártó BZ, Gaspari AA. Early immune events in the induction of allergic contact dermatitis. Nat Rev Immunol. 2012;12:114–124. 10.1038/nri3150 22240625 PMC3578582

[16] Albanesi C, Scarponi C, Cavani A, Federici M, Nasorri F, Girolomoni G. Interleukin-17 is produced by both Th1 and Th2 lymphocytes, and modulates interferon-gamma- and interleukin-4-induced activation of human keratinocytes. J Invest Dermatol. 2000;115:81–87. 10.1046/j.1523-1747.2000.00041.x 10886512

[17] Nakae S, Komiyama Y, Nambu A, Sudo K, Iwase M, Homma I, et al Antigen-specific T cell sensitization is impaired in IL-17-deficient mice, causing suppression of allergic cellular and humoral responses. Immunity. 2002;17:375–387. 12354389 10.1016/s1074-7613(02)00391-6

[18] Christensen AD, Haase C. Immunological mechanisms of contact hypersensitivity in mice. APMIS. 2012;120:1–27. 10.1111/j.1600-0463.2011.02832.x 22151305

[19] Cua DJ, Tato CM. Innate IL-17-producing cells: the sentinels of the immune system. Nat Rev Immunol. 2010;10:479–489. 10.1038/nri2800 20559326

[20] Jiang X, Clark RA, Liu L, Wagers AJ, Fuhlbrigge RC, Kupper TS. Skin infection generates non-migratory memory CD8+ T(RM) cells providing global skin immunity. Nature. 2012;483:227–231. 10.1038/nature10851 22388819 PMC3437663

[21] Jiang X, Campbell JJ, Kupper TS. Embryonic trafficking of gammadelta T cells to skin is dependent on E/P selectin ligands and CCR4. Proc Natl Acad Sci U S A. 2010;107:7443–7448. 10.1073/pnas.0912943107 20368416 PMC2867765

[22] Gebhardt T, Wakim LM, Eidsmo L, Reading PC, Heath WR, Carbone FR. Memory T cells in nonlymphoid tissue that provide enhanced local immunity during infection with herpes simplex virus. Nat Immunol. 2009;10:524–530. 10.1038/ni.1718 19305395

[23] Masopust D, Choo D, Vezys V, Wherry EJ, Duraiswamy J, Akondy R, et al Dynamic T cell migration program provides resident memory within intestinal epithelium. J Exp Med. 2010;207:553–564. 10.1084/jem.20090858 20156972 PMC2839151

[24] Wakim LM, Woodward-Davis A, Bevan MJ. Memory T cells persisting within the brain after local infection show functional adaptations to their tissue of residence. Proc Natl Acad Sci U S A. 2010;107:17872–17879. 10.1073/pnas.1010201107 20923878 PMC2964240

[25] Klonowski KD, Williams KJ, Marzo AL, Blair DA, Lingenheld EG, Lefrançois L. Dynamics of blood-borne CD8 memory T cell migration in vivo. Immunity. 2004;20:551–562. 15142524 10.1016/s1074-7613(04)00103-7

[26] Gebhardt T, Whitney PG, Zaid A, Mackay LK, Brooks AG, Heath WR, et al Different patterns of peripheral migration by memory CD4+ and CD8+ T cells. Nature. 2011;477:216–219. 10.1038/nature10339 21841802

[27] Watanabe R, Gehad A, Yang C, Scott LL, Teague JE, Schlapbach C, et al Human skin is protected by four functionally and phenotypically discrete populations of resident and recirculating memory T cells. Sci Transl Med. 2015;7:279ra39 10.1126/scitranslmed.3010302 25787765 PMC4425193

[28] Honda T, Egawa G, Grabbe S, Kabashima K. Update of immune events in the murine contact hypersensitivity model: toward the understanding of allergic contact dermatitis. J Invest Dermatol. 2013;133:303–315. 10.1038/jid.2012.284 22931926

[29] He D, Wu L, Kim HK, Li H, Elmets CA, Xu H. IL-17 and IFN-gamma mediate the elicitation of contact hypersensitivity responses by different mechanisms and both are required for optimal responses. J Immunol. 2009;183:1463–1470. 10.4049/jimmunol.0804108 19553527 PMC3179907

[30] Fink DR, Holm D, Schlosser A, Nielsen O, Latta M, Lozano F, et al Elevated numbers of SCART1+ gammadelta T cells in skin inflammation and inflammatory bowel disease. Mol Immunol. 2010;47:1710–1718. 10.1016/j.molimm.2010.03.002 20381152

[31] Krstic A, Mojsilovic S, Jovcic G, Bugarski D. The potential of interleukin-17 to mediate hematopoietic response. Immunol Res. 2012;52:34–41. 10.1007/s12026-012-8276-8 22392050

[32] Cai Y, Xue F, Fleming C, Yang J, Ding C, Ma Y, et al Differential developmental requirement and peripheral regulation for dermal Vγ4 and Vγ6T17 cells in health and inflammation. Nat Commun. 2014;5:3986 10.1038/ncomms4986 24909159 PMC4068267

[33] Malhotra N, Narayan K, Cho OH, Sylvia KE, Yin C, Melichar H, et al A network of high-mobility group box transcription factors programs innate interleukin-17 production.Immunity. 2013;38:681–693. 10.1016/j.immuni.2013.01.010 23562159 PMC3811080

[34] Sheridan BS, Lefrançois L. Regional and mucosal memory T cells. Nat Immunol. 2011;12:485–491. 21739671 10.1038/ni.2029 PMC3224372

[35] Park CO, Kupper TS. The emerging role of resident memory T cells in protective immunity and inflammatory disease. Nat Med. 2015;21:688–697. 10.1038/nm.3883 26121195 PMC4640452

[36] Weber FC, Németh T, Csepregi JZ, Dudeck A, Roers A, Ozsvári B, et al Neutrophils are required for both the sensitization and elicitation phase of contact hypersensitivity.J Exp Med. 2015;212:15–22. 10.1084/jem.20130062 25512469 PMC4291534

[37] Sutton CE, Lalor SJ, Sweeney CM, Brereton CF, Lavelle EC, Mills KH. Interleukin-1 and IL-23 induce innate IL-17 production from gammadelta T cells, amplifying Th17 responses and autoimmunity. Immunity. 2009;31:331–341 10.1016/j.immuni.2009.08.001 19682929

