# Supplementary figures and images for: Dermal γδ T Cells Do Not Freely Re-Circulate Out of Skin and Produce IL-17 to Promote Neutrophil Infiltration during Primary Contact Hypersensitivity
Source: PLoS One. 2017 Jan 12;12(1):e0169397. doi: 10.1371/journal.pone.0169397 (PMC5230790; doi:10.1371/journal.pone.0169397)

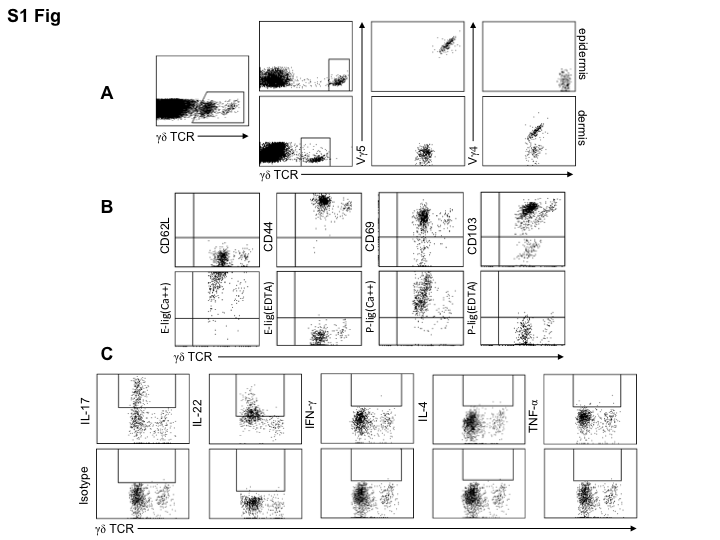

Supplement: S1 Fig — The ears of normal naive C57BL/6 mice at the age of 8–12 weeks were harvested and separated into dorsal and ventral sheets. Sheets were then chopped into small pieces and digested with RPMI 1640 supplemented with 5% FCS, 1% P/S, 1 mg/ml Collegenase D and 40 μg/ml DNase I at 37°C for 1 hour. In some cases, dorsal sheets were floated dermis side down on a 5% dispase solution for 20–30 min. Epidermis and dermis were gently separated and chopped into small pieces for digestion. Digested skin tissues were then mashed through a 70-μm nylon cell strainer to collect cell suspensions. After washed thoroughly with cold PBS, cells were stained with fluorescence-conjugated antibodies (A and B). E- or P-selectin ligands (E- or P-lig) were detected by CD62E/Fc or CD62P/Fc chimera in a calcium-dependent binding manner, respectively. C, skin cells were incubated with DMEM (10%FCS) containing 50 ng/ml PMA and 1 mM Ionomycin in the presence of Brefeldin A at 37°C for 6 hours. Cytokine productions of skin γδ T cells were measured by intracellular staining. Results are representative of at least three independent experiments. (TIFF) [file pone.0169397.s001.tiff]

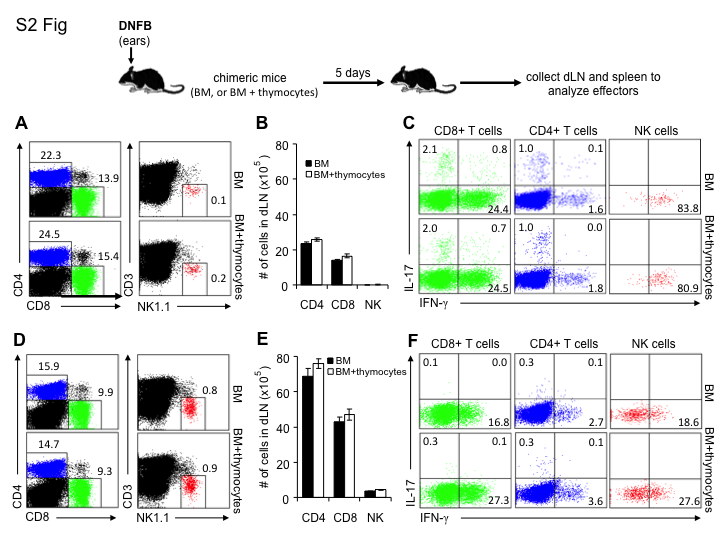

Supplement: S2 Fig — The ears of chimeric mice were sensitized with 0.25% DNFB for 2 consecutive days. 5 days later, draining lymph node (dLN) and spleen were harvested and CD4+ or CD8+ T cells and NK cells as well as their IL-17 / IFN-γ productions (measured as described at Fig 1) were analyzed by flow cytometry. dLN: A (percentage), B (cell numbers), and C (cytokine productions); spleen: D (percentage), E (cell numbers), and F (cytokine productions). Results are representative of two independent experiments. (TIFF) [file pone.0169397.s002.tiff]
